# Fatty acid profiles and their distribution patterns in microalgae: a comprehensive analysis of more than 2000 strains from the SAG culture collection

**DOI:** 10.1186/1471-2229-11-124

**Published:** 2011-09-06

**Authors:** Imke Lang, Ladislav Hodac, Thomas Friedl, Ivo Feussner

**Affiliations:** 1 Georg-August-University, Albrecht-von-Haller-Institute for Plant Sciences, Department of Plant Biochemistry, Göttingen, Germany; 2 Cyano-Biofuels GmbH, Magnussstrasse 11, 12489 Berlin, Germany; 3 Georg-August-University, Albrecht-von-Haller-Institute for Plant Sciences, Department of Experimental Phycology and Culture Collection of Algae in Göttingen (EPSAG), Göttingen, Germany

## Abstract

**Background:**

Among the various biochemical markers, fatty acids or lipid profiles represent a chemically relatively inert class of compounds that is easy to isolate from biological material. Fatty acid (FA) profiles are considered as chemotaxonomic markers to define groups of various taxonomic ranks in flowering plants, trees and other embryophytes.

**Results:**

The fatty acid profiles of 2076 microalgal strains from the culture collection of algae of Göttingen University (SAG) were determined in the stationary phase. Overall 76 different fatty acids and 10 other lipophilic substances were identified and quantified. The obtained FA profiles were added into a database providing information about fatty acid composition. Using this database we tested whether FA profiles are suitable as chemotaxonomic markers. FA distribution patterns were found to reflect phylogenetic relationships at the level of phyla and classes. In contrast, at lower taxonomic levels, e.g. between closely related species and even among multiple isolates of the same species, FA contents may be rather variable.

**Conclusion:**

FA distribution patterns are suitable chemotaxonomic markers to define taxa of higher rank in algae. However, due to their extensive variation at the species level it is difficult to make predictions about the FA profile in a novel isolate.

## Background

The analysis of the overall fatty acid profiles as well as the occurrence of fatty acids (FAs) in different lipid classes in microalgae is an emerging field which is expected to reveal the identification of novel FAs with a variety of new functional groups [1]. Despite a number of reports has been carried out and published, describing the contents as well as the composition of polyunsaturated fatty acids (PUFAs) in mostly marine microalgae [2-4], systematic approaches that include different or even many genera of microalgae and particularly those from freshwaters or terrestrial habitats are still missing [5].

Based on current knowledge, FA composition divides microalgae roughly into two groups, i.e. on one hand the cyanobacteria and green algae (Chlorophyta and Streptophyta) which contain low amounts of FAs, predominantly saturated and mono unsaturated FAs as well as trace amounts of PUFAs, mostly linoleic acid (LA, 18:2(9*Z*, 12*Z*): where x:y(z) is a fatty acid containing X carbons and y double bonds in position z counting from the carboxyl end)). On the other hand Chromalveolate algae contain significant amounts of PUFAs [6].

Among the various biochemical markers, FA or lipid profiles represent a chemically relatively inert class of compounds that is easy to isolate from biological material and FA profiles are considered as chemotaxonomic markers to define groups of various taxonomic ranks in flowering plants, trees and other Embryophytes [7,8].

Beside the identification of novel FAs, some recent studies report on the use of FAs and lipid profiles of algae as biomarkers [1,9-11]. Viso et al. determined profiles of FAs of nine different marine algal groups and they were able to define even species-specific lipid compositions [4]. Moreover they found a roughly taxon specific profile when the cells were cultured under identical growth conditions. Various strains and species of the cyanobacterium *Nostoc *were screened for their FA content and the application of a FA-based cluster analysis has been described for their identification [12].

FA and lipid composition have also been used as biomarkers to distinguish closely related microalgae at the species and the generic levels [11,13]. Hitherto no systematic analysis has been carried out on a large scale basis on either the profiles of lipids or FAs in microalgae. Therefore, we determined the FA profiles of all available microalgal strains of the SAG culture collection of microalgae http://www.epsag.uni-goettingen.de which is one of the most diverse and comprehensive resources of microalgae. At present (March 2011) 2291 strains of mainly microscopic algae including a considerable variety of cyanobacteria is available. They comprise almost all phyla and classes of eukaryotic algae, but an emphasis is put on algae from freshwaters and terrestrial habitats.

Distribution patterns of FAs may be valuable also as a proxy to identify certain groups, species and strains of microalgae of particular interest for applied research, i.e. due to the presence of certain FAs and/or high percentages of total FA content. We also tested whether the detected FA distribution patterns are meaningful in a phylogenetic context at various taxonomic levels, i.e. to define taxonomic groups of microalgae by their FA patterns. It would assist predicting FA content and/or presence of other valuable compounds if the phylogenetic relationships of algae were reflected in their FA distribution patterns.

Here the focus was set on esterified long chain FAs (C-14 - C-24), which were analysed via Gas chromatography (GC) with or without mass spectrometry (MS). The large number of data obtained, were added into a database to document the FA profiles of the studied microalgal strains.

## Results and Discussion

### 1. A database of FA profiles from diverse microalgae

The characterisation of FA profiles of the SAG microalgal strains was performed by screening long chain FAs (C-14 - C-24) esterified within lipids. A total of 2076 culture strains from the SAG (equal 91% of the SAG's holding) were screened. A database was established which contained all identified FAs and some other hydrophobic metabolites. An overview of all substances identified in the algal strains screened is shown in Table 1. A total of 86 different substances were identified by mass spectrometry, 76 of which represent methyl esters of FAs. Out of the 76 fatty acids, 36 substances were identified by their mass spectrum and by retention time according to a standard substance, and the other 40 fatty acids were identified by their mass spectra only. The remaining 10 substances were identified by their mass spectra only as well. In comparisons with a standard substance, the compound was identified by comparison to mass spectra with highest similarity to the proposed substance in the MS-library (Nist02 or Wiley98). By this some methyl esters of branched FAs were detected, for example 12-methyl-14:0 or 3, 7, 11, 15-tetramethyl-16:0. Whereas for most of the FAMEs, authentic standards or MS references were available, for some other substances only "best hit" identification was possible. The DMOX derivatives enabled the identification of the remaining 12 FAMEs. Unidentified substances have yet to be verified with authentic standards, which are not available at this time point. The complete database is shown as additional file 1.

**Table 1:** Overview of the FAMEs identified and other substances found in the analysed SAG microalgal strains

86 substances, 76 methyl esters of FAs		
**methyl esters of saturated straight-chain FAs**	**methyl esters of branched chain FAs**	**methyl esters of monoenoic FAs**

14:0	12-methyl-14:0	14:1 (7*Z*)
16:0	13-methyl-14:0	14:1 (9*Z*)
17:0	14-methyl-15:0	15:1 (10*Z*)
18:0	14-methyl-16:0	16:1 (5*Z*)
19:0	methyl-3, 7, 11, 15-tetramethyl-16:0	16:1 (7*Z*)
20:0	16- o. 15-methyl-17:0	16:1 (9*Z*)
21:0	17-methyl-18:0	16:1 (11*Z*)
22:0	6, 10, 14 trimethyl-2-pentadecanone	17:1 (8*Z*)
23:0		17:1 (9*Z*)
24:0		17:1 (10*Z*)
		18:1 (9*E*)
**methyl esters of dienoic FAs**	**methyl esters of trienoic FAs**	18:1 (9*Z*)
15:2	16:3 (4*Z*, 7*Z*, 10*Z*)	18:1 (11*Z*)
16:2 (7*Z*, 10*Z*)	16:3 (6*Z*, 9*Z*, 12*Z*)	19:1 (11*Z*)
16:2 (9*Z*, 12*Z*)	16:3 (7*Z*, 10*Z*, 13*Z*)	20:1 (11*Z*)
17:2 (7*Z*, 10*Z*)	17:3	22:1 (13*Z*)
17:2 (9*Z*, 12*Z*)	18:3 (5*Z*, 9*Z*, 12*Z*)	24:1 (15*Z*)
18:2 (6*Z*, 9*Z*)	18:3 (6*Z*, 9*Z*, 12*Z*)	
18:2 (8*Z*, x*Z*)*	18:3 (8*Z*, 11*Z*, 14*Z*)	
18:2 (9*E*, 12*E*)	18:3 (9*Z*, 12*Z*, 15*Z*)	
18:2 (9*Z*, 12*Z*)	19:3	
18:2 (9*Z*, 14*Z*)	19:3	
18:2 (11*Z*, 14*Z*)	20:3 (7*Z*, 10*Z*, 13*Z*)	
19:2 (9*Z*, 12*Z*)	20:3 (8*Z*, 11*Z*, 14*Z*)	
20:2 (11*Z*, 14*Z*)	20:3 (11*Z*, 14*Z*, 17*Z*)	
22:2 (13*Z*, 16*Z*)	22:3	
		
**methyl esters of tetra-, penta-, and hexaenoic FAs**	**other substances**	
16:4 (4*Z*, 7*Z*, 10Z, 13Z)	(8*Z*, 11*Z*)-heptadeca-8, 11-dienal	
16:4 (6*Z*, 9*Z*, 12*Z*, 15*Z*)	3-(3, 5-ditertbutyl-4-hydroxyphenyl) propionate	
18:4 (5*Z*, 9Z, 12*Z*, 15*Z*)	3, 7, 11, 15-tetramethyl-2-hexadecen-1-ol	
18:4 (6*Z*, 9*Z*, 12*Z*, 15*Z*)	8-(2-octylcyclopropyl) octadecanoate	
19:4	2, 3, 4, 5- tetramethyl-3-hexen	
20:4 (5*Z*, 8*Z*, 11*Z*, 14*Z*)	(5*Z*, 8*Z*, 11*Z*)-15, 16 epoxy 5, 8, 11-octadecadienoate	
20:4 (8*Z*, 11*Z*, 14*Z*, 17*Z*)	Tetradecanamide	
22:4 (7*Z*, 10*Z*, 13*Z*, 16*Z*)	Hexadecanamide	
18:5 (3*Z*, 6*Z*, 9*Z*, 12*Z*, 15*Z*)	(9*Z*)-Octadecenamide	
20:5 (5*Z*, 8*Z*, 11*Z*, 14*Z*, 17*Z*)	9, 10-methylene tetradecanoate	
22:5 (4*Z*, 7*Z*, 10*Z*, 13*Z*, 16*Z*)		
22:5 (7*Z*, 10*Z*, 13*Z*, 16*Z*, 19*Z*)		
22:6 (4*Z*, 7*Z*, 10*Z*, 13*Z*, 16*Z*, 19*Z*)		

For the marked (*) FAMEs the double bond positions were only tentatively assigned.

Bacteria in algal cultures (as contaminations or sometimes even through symbiosis) are well known and can be found in culture strains of almost any algal culture collection. Only a small fraction (about 20%) of the studied SAG strains may be in axenic state. Therefore, also the FA content of the contaminating bacteria may have contributed to the obtained FA profile. To test this, we measured methyl-15:0 and methyl 17:0 that are regarded as markers for bacterial contaminations [4]. Only 34 strains out of the 2076 analyzed strains contained small amounts methyl-15:0. This observed low rate of contaminating bacteria was supported by microscopic controls which are routine in the perpetual maintenance of algal strains (data not shown). In summary, we conclude that only 1-2% of the strains may have been contaminated and that there is only a minor influence of bacterial contaminations on the observed algal culture FA profiles.

In addition we compared the measured major FA profiles of 10 randomly chosen strains from different classes with published data (Table 2), and it should be noted that only one out of the 10 strains that were chosen from the published data originated from the SAG collection. For 6 strains the FA profiles were very similar. In case of the 4 remaining strains major differences were observed in the degree of desaturation of the FAs with different chain lengths, which may be explained by the different cultivation conditions used in the different studies.

**Table 2:** Comparison of the major FA composition of algae observed in this study against data published previously

Species	FA	(%	of	total)											Ref
		
	14:0	16:0	16:1	16:2	16:3	16:4	18:0	18:1	18:2	18:3	18:4	20:4	20:5	22:6	
**Bacillariophyceae**															
Phaeodactylum	9.2	26.8	45.4	-	-	-	0.7	4.6	-	-	-	-	12.3	1.1	a
tricornutum	9.4	23.7	35.8	-	-	-	6.0	3.3	4.4	3.2	0.2	-	13.3	0.9	b
	6.7	14.7	43.6	2.0	-	-	-	15.8	0.5	0.4	1.1	-	14.4	0.7	e
Thalassiosira weissflogii	25.9	28.8	28.7	-	-	7.4	1.5	3.3	-	0.3	-	-	4.0	0.1	b
	8.8	36.6	40.5	-	-	-	-	14.0	-	-	-	-	-	-	e
**Chlorophyceae**															
Dunaliella primolecta	0.4	21.8	4.5	0.9	2.5	12.3	0.8	6.4	6.2	41.1	4.1	-	-	-	b
	0.6	26.0	0.9	-	-	-	1.6	16.3	7.0	38.7	0.6	-	-	-	e
Nannochloris sp.	1.8	15.1	16.6	-	0.2	-	1.0	57.7	0.6	0.8	0.3	5.9	-	-	b
	13.3	17.8	-	-	-	-	-	23.9	10.8	28.2	6.1	-	-	-	e
Parietochloris incisa	-	10.0	2.0	1.0	1.0	-	3.0	16.0	17.0	3.0	-	46.0	1.0	-	c
	0	19.8	-	5.2	-	-	18.2	10.2	14.3	14.3	-	14.0	4.3	-	e
**Cyanophyceae**															
Nostoc commune	0.3	43.5	11.3	0.4	-	-	1.5	6.9	19.3	16.3	-	-	-	-	d
	-	25.3	24.1	-	-	-	-	-	12.5	38.1	-	-	-	-	e
Synechocystis sp.	13.4	26.5	43.6	-	-	-	3.5	8.0	0.2	4.7	-	-	-	-	b
	42.5	18.8	30.1	-	-	-	-	-	-	14.2	-	-	-	-	e
**Haptophyceae**															
Pavlova lutheri	11.8	23.6	28.3	-	-	-	2.0	12.4	-	-	-	-	12.1	9.7	a
	10.1	11.1	26.3	-	-	-	-	5.2	0.6	0.5	9.1	0.3	18.0	9.7	e
**Prymnesiophyceae**															
Emiliana huxleyi	41.7	17.7	5.5	-	-	-	2.1	21.7	0.9	5.5	5.0	-	-	-	b
	18.8	10.3	-	-	-	-	10.8	42.2	-	-	8.7	-	-	9.2	e
**Raphidophyceae**															
Heterosigma akashiwo	6.2	46.3	21.3	-	-	0.4	0.5	2.7	1.6	4.2	7.3	-	8.7	0.7	b
	6.6	40.0	12.7	4.0	-	-	-	-	4.5	6.7	5.2	3.5	14.8	-	e

a [3]

b [4]

c [20]

d [12]

e this work

### 2. Patterns of fatty acid composition

**Figure 1:**
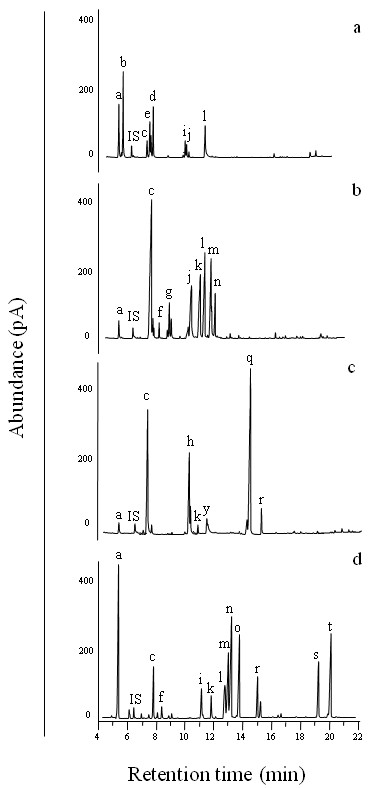
**Representative gas chromatograms of fatty acid methyl esters from four species belonging to different algal groups**. a) Cyanobacteria, *Chroococcus minutus *SAG 41.79; b) Chlorophyta, *Closteriopsis acicularis *SAG 11.86; c) Rhodophyta, *Pseudochantransia *spec. SAG 14.96; d) Chromalveolates (Haptophyta), *Prymnesium parvum *SAG 127.79. Fatty acid methyl esters: a) 14:0, b) 14:1n-5, c) 16:0, d) 16:1n-9, e) 16:1n-7, f) 16:2n-6, g) 16:4n-3, h) 18:0, i) 18:1n-9, j) 18:1n-7, k) 18:2n-6, l) 18:3n-6, m) 18:3n-3, n) 18:4n-3, o) 18:5n-3, p) 20:3n-6, q) 20:4n-6, r) 20:5n-3, s) 22:5n-3, t) 22:6n-3.

FAME profiles were rather different among strains. As an example, FAME profiles from four different genera, i.e. *Chroococcus *(Cyanobacteria), *Closteriopsis *(Chlorophyta, Trebouxiophyceae), *Pseudochantransia *(Rhodophyta) and *Prymnesium *(Chromalveolates, Haptophyta) are presented in Figure 1. Therefore it was anticipated to recover certain different FA distribution patterns between phyla, classes and genera of microalgae. In addition, it was tested whether differences in FA patterns can also be found for groups at lower taxonomic rank, i.e. between species of the same genus or even among multiples isolates of the same species.

#### 2.1 Distribution of four important PUFAs among strains of the SAG algal culture collection

The distribution patterns of FAs among and within the 17 groups (phyla or classes) of microalgae and the cyanobacteria comprised by the examined strains was investigated in more detail for four PUFAs which are of high nutritional interest (Table 3). The frequency of occurrence of these four PUFAs in a certain group of microalgae is given as the percentage of strains with a certain FA from all examined strains in Table 3.

**Table 3:** Frequency of four selected PUFAs in 17 taxonomic groups of microalgae on which the examined 2071 strains of the SAG culture collection were distributed, and the size of each group (in total number of strains)

			no. of strains	DHA	EPA	ARA	GLA
Cyanobacteria			223	1.3	0.9	0.4	12.1
Plantae	Glaucophyta		15		80.0	46.7	6.7
	Chlorophyta	Chlorophyceae	927	5.1	6.9	5.7	26.2
		Trebouxiophyceae	253	4.3	16.6	22.9	6.3
		Ulvophyceae	70	4.3	22.9	12.9	7.1
		prasinophytes	21	14.3	33.3	42.9	57.1
	Charophyta		159	1.3	17.6	13.8	31.4
	Rhodophyta		78		70.5	67.9	3.8
Excavates	Euglenoids		131	42.7	44.3	51.1	
Chromalveolates	Stramenopiles	Bacillariophyceae	18	22.2	44.4	11.1	11.1
		Xanthophyceae	81	4.9	75.3	49.4	16.1
		Eustigmatophyceae	17		88.2	41.2	5.9
		Phaeophyceae	12		58.3	91.7	16.7
		Chryso-/Synurophyceae	12	16.7	33.3	8.3	16.7
	Haptophyta		13	84.6	61.5	7.7	
	Cryptophyta	Cryptophyta	27	22.2	66.7	3.7	3.7
	Alveolates	Dinophyta	14	64.3	57.1	14.3	

			2071				

The frequency of PUFAs is shown as the percentage of the total number of strains examined per group.

Because the SAG culture collection focuses on microscopic algae from terrestrial habitats, the Haptophyta, Dinophyta and Phaeophyceae were just poorly represented. Therefore, the recovered distribution patterns in these and other poorly represented groups may not be representative for the whole group. For instance, for Phaeophyceae mainly microscopic forms (e.g., *Ectocarpus *and the freshwater genus *Bodanella*) were available and the examined Rhodophyta strains covered mostly freshwater forms or those from terrestrial habitats (e.g., *Porphyridium*). Although diatoms are very diverse in terrestrial habitats, the examined small sample of available diatom strains (18) does by far not adequately represent this group which is probably the most species-rich algal group. Also, for each of the two classes of Stramenopiles (heterokont algae), Phaeothamniophyceae and Raphidophyceae, just two strains are maintained at the SAG and, therefore, are not further discussed here. Similarly, there is only a single strain of Chlorarachniophyta (Rhizaria supergroup) in the SAG.

The very long chain PUFA docosahexaenoic acid (DHA, 22:6(4*Z*, 7*Z*, 10*Z*, 13*Z*, 16*Z*, 19*Z*)) was the third most frequent FA, present in 15 out of 20 examined groups (Table 3). In the Dinophyta, Haptophyta and Euglenoids DHA-containing strains were particularly frequent and DHA was found there in relatively high percentages of total FA content, i.e. in 60% or more of these strains the DHA proportion was higher than 5%. In the single studied dinophyte strain of *Ceratium horridum *the DHA proportion was even 29.3%. In the other groups DHA was found in rather low frequencies and also mostly in rather small proportions, i.e. less than 1% of total FA content. Although DHA was found in the Cryptophyta and Bacillariophyceae in about every fifth strain, its percentage of total FA content was less than 5% there, except in *Cryptomonas baltica *SAG 18.80 (Cryptophyta) where it is was 13.7%. Despite DHA was found in rather low frequencies in the green algae (Chlorophyta), the second highest DHA content of all SAG strains, 18.9% of total FA, was found in the chlorophyte *Chlorococcum novae-angliae *SAG 5.85, followed by the trebouxiophyte *Prototheca zopfii *SAG 263-8 with 14.2%. Together these findings are in accordance with DHA amounts described before for specific groups of alga [3,4,14,15].

Eicosapentaenoic acid (EPA, 20:5(5*Z*, 8*Z*, 11*Z*, 14*Z*, 17*Z*)) was one of the most common PUFAs, found in all of the 17 groups covered by our study (Table 3). EPA-containing strains were particularly frequent in the Eustigmatophyceae, Glaucophyta, Xanthophyceae and Rhodophyta. The highest EPA proportions of total FA content were in the Rhodophyta, with about 81% of the strains exhibiting more than 10% EPA. The highest values were 52.4% in *Compsopogonopsis leptoclados *SAG 106.79 and 44.9% in *Acrochaetium virgatulum *SAG 1.81. Also strains of three species of *Porphyridium *contained high amounts of EPA (31.2% in *P. sordidum *SAG O 500, 27.5% in *P. aerugineum *SAG 110.79, 26.7% in *P. purpureum *SAG 1380-1a). This is in agreement with a report on *P. cruentum *suggesting that red algae are a rich source of EPA [16]. Despite EPA was rather frequently found in the Glaucophyta, only about half of all strains had EPA proportions greater than 10% (maximum 31.1% in *Glaucocystis nostochinearum *SAG 28.80). This is in agreement with another study which showed high amounts of EPA (besides ARA) in the glaucophyte *Cyanophora paradoxa *[17]. The highest percentage (87%) of strains with an EPA proportion of greater than 10% was in the Dinophyta, but with a maximum of just 24.3% in *Pyrocystis lunula *SAG 2014. In the Euglenoids, Xanthophyceae and Eustigmatophyceae about 67% of all strains had an EPA proportion of greater than 10% with maximum values of about 31% (31.4% in *Heterococcus fuornensis *SAG 835-5, 31.6% in *Euglena proxima *SAG 1224-11a) and 34.6% in *Goniochloris sculpta *SAG 29.96. EPA was rarely found and mostly in insignificant amounts (< 5%) in most green algae, but three strains had an exceptionally higher content of about 20% of total FAs (24.2%, *Chlorella *sp. SAG 242.80; 24.0%, *Chlamydomonas allensworthii *SAG 28.98; 22.3%, *Cylindrocapsa involuta *SAG 314-1). EPA was the only FA recovered from *Chlorarachnion repens *SAG 26.97 (Chlorarachniophyta). That Xanthophyceae and Eustigmatophyceae contain EPA in relatively high proportions while green algae rarely accumulate EPA supports previous studies [3,4,14,15,18].

Arachidonic acid (ARA, 20:4(5*Z*, 8*Z*, 11*Z*, 14*Z*)) was most frequently found in the Phaeophyceae where it was present in all strains except one investigated strain (Table 3); in about 54% of all Phaeophyceae strains the proportion of ARA was higher than 10%, but with a maximum of just 17.7% in *Halopteris filicina *SAG 10.96. ARA had the highest proportion of total FA in the Rhodophyta; there even about 77% of all strains had an ARA content of more than 10% with a maximum of 68.3% in *Pseudochantransia *sp. SAG 19.96. Interestingly, the ARA content was rather high but variable among the eight examined multiple isolates of the rhodophyte *Porphyridium purpureum*. While the average ARA proportion was about 31% in six strains, it was just 3.8% in SAG 1380-1d, but 44.5% in SAG 1380-1e. We have no explanation for this variation yet; both strains were isolated from marine habitats and are kept under the same culture conditions. High proportions of ARA (as well as EPA) were already found characteristic of another species of *Porphyridium cruentum *[16]. ARA was present in about half of all investigated Euglenoid strains and with relatively high proportions of total FA content, i.e. about one third of the strains exhibited more than 5% ARA with extraordinarily high values of 41.3% and 34.3% in *Rhabdomonas incurva *SAG 1271-8 and *Khawkinea quartana *SAG 1204-9. Interestingly, another strain of the same species *K. quartana*, SAG 1204-9, had less than half (13.3%) of ARA content and in five other species of *Rhabdomonas *no ARA was detected. This demonstrates that FA contents may be rather variable between species of the same genus and even among multiple isolates of the same species. Although about half of all examined strains for the Xanthophyceae and Eustigmatophyceae contained ARA (Table 3), they had this FA in relatively low proportions. Only one fourth of the ARA-containing Xanthophyceae strains exhibited more than 5% and in the Eustigmatophyceae even no strain reached 5%. ARA was rarely found in the green algae, i.e. with an average frequency of about 14% in the phyla Chlorophyta and Streptophyta, except for prasinophyte green algae where ARA was present in 42.9% of all strains (Table 3). However, there were a few single green algal examples with extraordinarily high ARA contents, i.e. 73.8% (corresponding to 102 μg/mg of dry weight, the highest ARA content detected in all investigated SAG strains) in the chlorophyte *Palmodictyon varium *SAG 3.92, followed by 52.9% in the chlorophyte *Trochisciopsis tetraspora *SAG 19.95 and 51.8% in the trebouxiophyte *Myrmecia bisecta *SAG 2043. That a high ARA content was found in the latter strain is in agreement with that it has been found a close relative with *Parietochloris incisa *(syn. *Lobosphaeropsis incisa, Myrmecia incisa*) [19]. *P. incisa *has been assigned an "oleaginous microalga" and the richest plant source of ARA known so far due to its capability to accumulate high amounts of ARA (up to 59% of its total FA content) [20]. Interestingly, the SAG strain of *P. incisa *(*Lobosphaera incisa *SAG 2007) had with 13.2% a much lower ARA content (Table 2).

γ-Linolenic acid (GLA, 18:3(6*Z*, 9*Z*, 12*Z*)) was the third most common FA in the studied sample of SAG microalgal strains, missing only in the Haptophyta, Dinophyta and Euglenoids (Table 3). It was most frequently detected in two lineages of green algae, the prasinophytes and the Streptophyta. In prasinophytes, however, GLA was present only in one out of five genera available for that group, *Tetraselmis*, and there in 12 out of the 17 available strains and with variable proportions, i.e. 0.5 - 7.3% of total FA content. In the Streptophyta, GLA was more widely distributed, i.e. it was detected in 17 out of 41 examined genera. GLA distribution was rather variable within strains and species of a certain streptophyte genus, similar to findings of ARA in other genera. Relatively high percentages of GLA were found in species/strains of *Closterium *(16.5% in *C. baillyanum *SAG 50.89, 8% in *C. lunula *SAG 7.84), but GLA was not found in the other 12 strains of that genus. Similarly, in the many strains available for *Cosmarium *(25) and *Micrasterias *(16), GLA was found in only 11 and 2 strains, respectively. The highest percentages of GLA were found in the green algal class Chlorophyceae (29.9% in *Deasonia multinucleata *SAG 25.95, 28.5% in *Desmodesmus multiformis *SAG 26.91) and in Cyanobacteria (24.8% in *Spirulina maxima *SAG 84.79). In about one third (32%) of all chlorophyte GLA strains this FA had precentages of 5% and higher. Distribution of GLA in the cyanobacteria was rather patchy, i.e. the 27 cyanobacteria strains with GLA were mainly restricted to three genera, *Calothrix *(8 strains), *Microcystis *(7 strains) and *Spirulina *(6 strains). Also within each of these genera the GLA percentages were quite variable, e.g. in *Spirulina *it varied from 4.6% to 24.8%, and three strains where without GLA. FA composition has previously been used to discriminate cyanobacteria in isolates and natural samples at the generic level [21,22]. To discriminate species of cyanobacteria, as an additional marker the hydrocarbon composition was used in an earlier study, but in our study we failed to detect any substance out of this group [23]. Interestingly, GLA was the only FA that was detected in more than three out of the 223 examined strains. Therefore, the SAG cyanobacteria strains may be roughly divided into those with GLA present (few genera) and those where almost no PUFAs were present. This corresponds to the earlier findings that described a bipartition of cyanobacteria, independent of their taxonomic position, into genera producing C-18 PUFA and those which do not [24,25].

The prasinophyte genus *Tetraselmis *presented an interesting example to test for FA variation among closely related isolates. Nine strains assigned to that genus have been isolated from the same (marine) locality and regarded as the same species by the isolator (U.G. Schlösser, pers. comm.). Only in two strains DHA was present, but in very small traces (0.3% and 0.4%). In contrast, ARA and GLA were found in all isolates with percentages varying from 0.8% to 2.7% and 0.5% to 7.3%, respectively.

#### 2.2 Analysis of FA distribution patterns

The detected fatty acid (FA) composition of the 2076 investigated strains was statistically analyzed to test whether certain patterns of FA distribution among the various investigated algal groups are present that may correspond to their phylogenetic relationships. In a first set of three analyses (higher taxonomic levels) it was tested 1) whether FA distribution patterns may reflect differences among algal phyla derived from primary (Plantae supergroup) or secondary endocytobiosis (Chromalveolates, Euglenoids) compared to cyanobacteria representing the plastid origin, 2) the distinction of phyla within the Plantae supergroup (Chlorophyta, Streptophyta, Rhodophyta/Glaucophyta) and 3) major evolutionary lineages (classes) within the Chlorophyta. A second set of analyses focused at the generic level, i.e.it was tested whether separation of genera as based on previous 18S rDNA sequence analyses suggested for *Chlamydomonas *s.l., *Chlorella *s.l. and *Scenedesmus *s.l. are reflected in the FA distribution patterns. For the first set of analyses the many species (266) which were represented as multiple strains (e.g., *Chlamydomonas moewusii*, 28) had to be reduced to only a single strain per species to avoid biases. This included also the multiple strains unidentified at the species level, i.e. labelled with "sp." instead a species name (e.g., *Chlorogonium *sp., 26). The SAG's Chlorophyta strains were particularly rich in such multiple strains. Also excluded were those strains where only a single FA was detected. This reduced the total number of strains considered in our calculations to 1193. The strains were then divided into eleven groups roughly corresponding to phyla or classes (Additional file 2). Strains belonging to the Chlorophyta (61% of all investigated strains) were further subdivided into the three classes, Chlorophyceae, Trebouxiophyceae, and Ulvophyceae, whereas the prasinophyte SAG green algal strains (1.7% of all considered Chlorophyta strains) were excluded from the analyses because they comprised only very few species (10). The strains of Glaucophyta (15) and Rhodophyta (81) were collectively treated as one composite unit. The Rhizaria - Chlorarachniophyta, was represented just by a single strain and, thus, was omitted from the statistical analyses.

##### Higher taxonomic levels analyses

**Figure 2:**
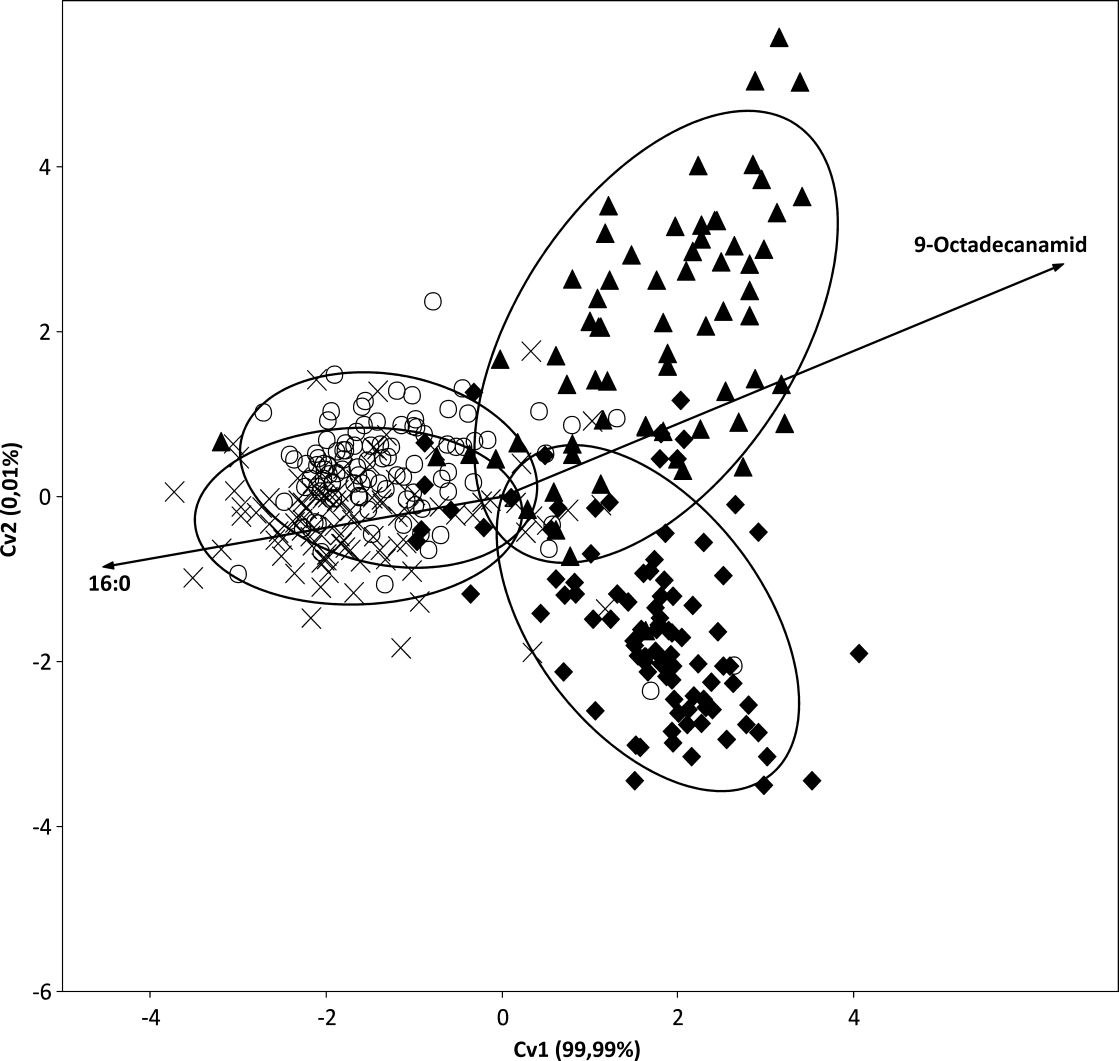
**Discrimination of cyanobacteria and three algal eukaryotic supergroups (Plantae, Chromalveolates, Excavates/Euglenoids) as based on fatty acid distribution patterns of 373 investigated cyanobacterial and algal strains using Canonical Variates Analysis**. The two vectors shown indicate FAs significantly correlated with canonical axis 1. Lines encircle 95% of members of a particular group. Circles, Cyanobacteria; crosses, Plantae; arrowheads, Excavates/Euglenoids; diamonds, Chromalveolates.

**Figure 3:**
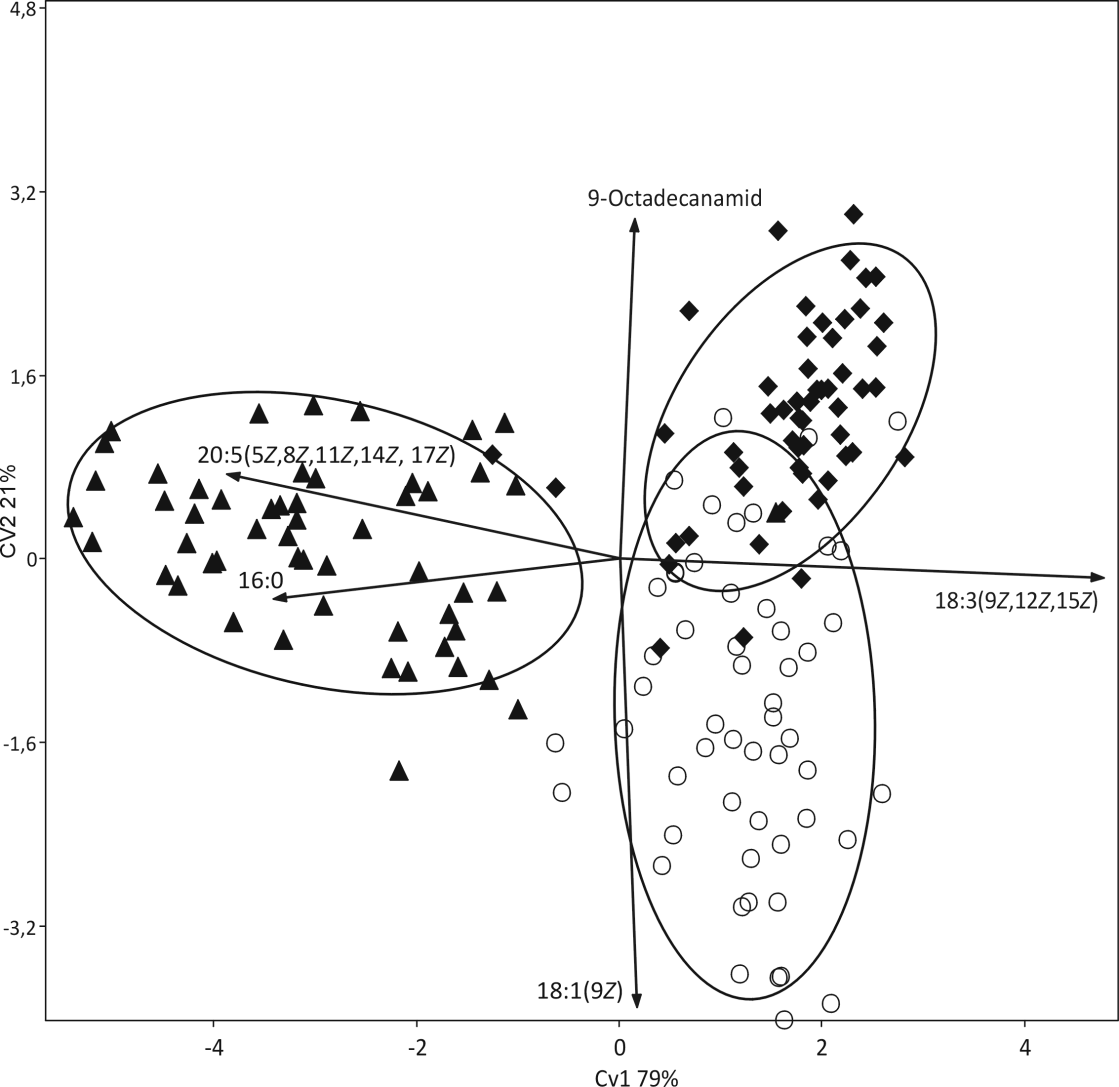
**Discrimination of 162 algal strains of the Plantae supergroup into three subgroups representing the Rhodophyta/Glaucophyta composite group (arrowheads) and both green algal phyla, Chlorophyta (diamonds) and Streptophyta (circles) as based on their fatty acid distribution patterns using Canonical Variates Analysis**. The vectors shown indicate FAs significantly correlated with CV1 and CV2. Lines encircle 95% of members of a particular group.

**Figure 4:**
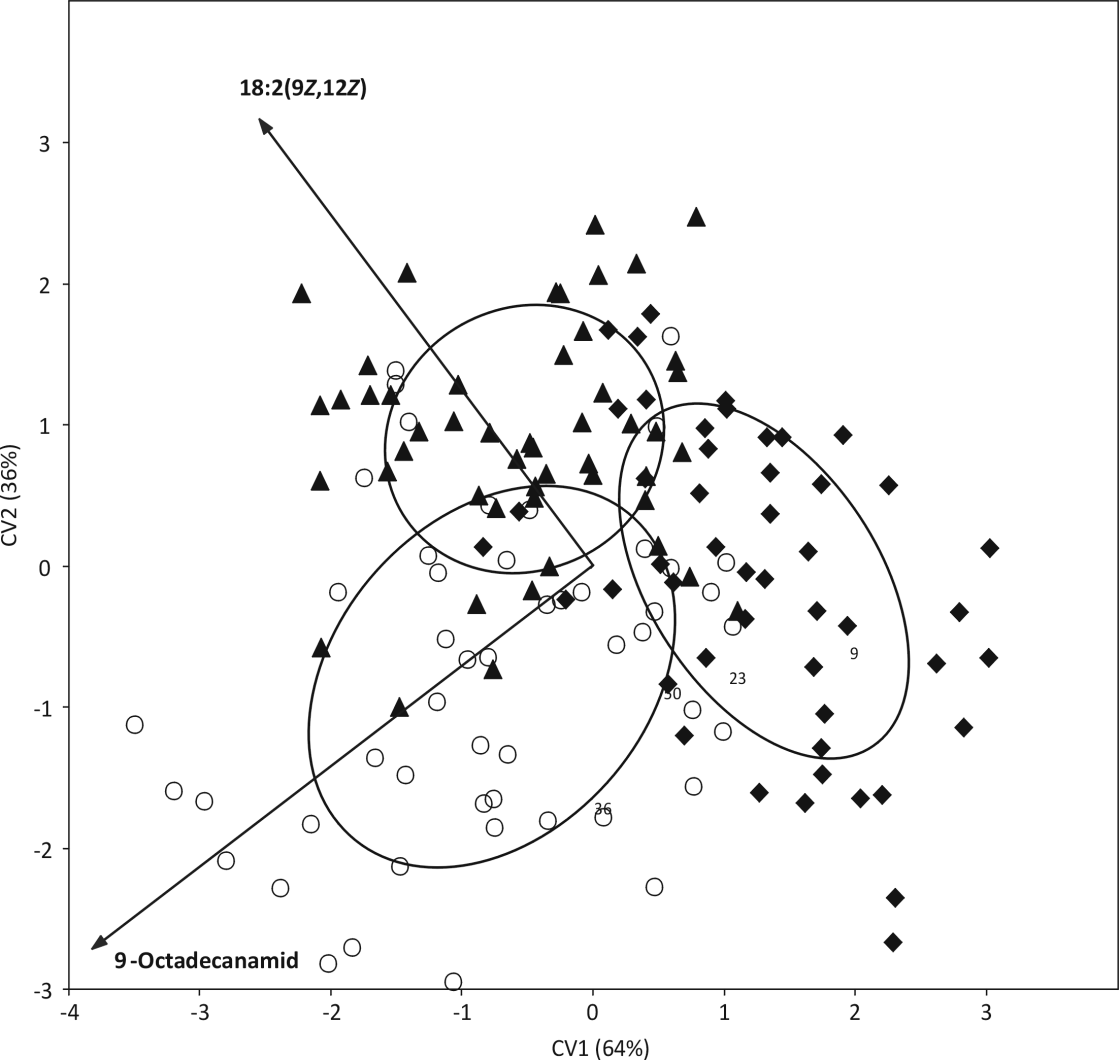
**Discrimination of 162 algal strains of the Chlorophyta into three subgroups representing the three green algal classes Chlorophyceae (diamonds), Trebouxiophyceae (arrowheads) and Ulvophyceae (circles) as based on their fatty acid distribution patterns using Canonical Variates Analysis**. Both vectors correspond to variables (fatty acids) correlated with both canonical axes. Lines encircle 65% of members of a particular group.

It was tested whether distribution patterns of FA composition on the investigated strains delineate the three "super groups" of eukaryotic algae, Plantae, Chromalveolates and Excavates (Euglenoids), and the cyanobacteria from each other. The Plantae super group comprises exclusively eukaryotes with plastids derived from primary endocytobiosis, i.e. a cyanobacterium was transformed into an organelle through uptake and retention by the host cell followed by the loss of much of its genome [26]. Chromalveolate algae as well as the Euglenoids (the only algal lineage of Excavates) acquired their plastids through secondary endocytobiosis from rhodophyte and a green alga, respectively [26,27]. To consider almost equal numbers of strains for all four groups, 100 strains of Plantae, Chromalveolates and Cyanobacteria were randomly selected which closely amounts the total number of considered euglenoid strains (73). The ordination which resulted from CVA (Canonical Variates Analysis, multigroup discriminant analysis) pointed out a strong difference between cyanobacteria/primary endocytobiosis (Plantae) and the two groups representing secondary endocytobiosis (Chromalveolates/Euglenoids) (Figure 2). The observed difference was without exception supported by non-parametric significance tests for multidimensional data (NP-MANOVA and ANOSIM). Following SIMPER, the lowest observed dissimilarity (63.55%) was between Cyanobacteria and Plantae, while the highest (77.29%) was between Plantae and Chromalveolates. The first canonical variate (CV1) involved 99.99% of all possible differences among the four groups, hence we examined for possible correlations between this axis and FAs. Four FAs were significantly and exclusively correlated with the first canonical variate (CV1), i.e. 16:0 (ρ_CV1 _= -0.61/p < 0.001), 18:2(9*Z*, 12*Z*) (ρ_CV1 _= -0.46/p < 0.001), 9-octadecanamid (ρ_CV1 _= 0.41/p < 0.001), and 18:1(9*Z*) (ρ_CV1 _= -0.17/p = 0.001). In a second analysis it was tested whether FA distribution patterns distinguish phyla of the Plantae super group, i.e. the two lineages of green algae, Chlorophyta and Streptophyta [28,29], and the composite Rhodophyta/Glaucophyta group. Because the latter was with 54 strains the smallest group, it was compared with equally large random samples from each the Chlorophyta and Streptophyta (Table 3). The ordination diagram from a CVA of the total of 162 investigated strains clearly separated the Rhodophyta/Glaucophyta group from both green algal phyla (Figure 3). CV1 involved 79% of all possible differences and even CV2 was with 21% not negligible. The significance tests, NP-MANOVA and ANOSIM, supported the distinction of all three groups. SIMPER showed the Rhodophyta/Glaucophyta composite group rather dissimilar from both green algal phyla, i.e. there were dissimilarities of 70.55% and 71.53% with the Chlorophyta and Streptophyta, respectively. The lowest dissimilarity (55.41%) among the three tested groups was between Chlorophyta and Streptophyta. There were five FAs significantly and exclusively correlated with CV1, i.e. 18:3(9*Z*, 12*Z*, 15*Z*) (ρ_CV1 _= 0.77/p < 0.001), 20:4 (ρ_CV1 _= -0.49/p < 0.001), 20:5(5*Z*, 8*Z*, 11*Z*, 14*Z*, 17*Z*) (ρ_CV1 _= -0.59/p < 0.001), 18:1(9*Z*) (ρ_CV1 _= 0.30/p = 0.001) and 16:0 (ρ_CV1 _= -0.56/p = 0, 001). Two FAs were correlated exclusively with CV2, i.e. they discriminated Chlorophyta and Streptophyta, 18:1(9*Z*) (ρ_CV2 _= -0.4477/p < 0.001) and 9-octadecanamid (ρ_CV2 _= 0.34/p < 0.001). The by far largest fraction of all considered strains (60.3%) were from the Chlorophyta which made it interesting to test whether FA distribution patterns can discriminate between the three classes of Chlorophyta, the Chlorophyceae, Trebouxiophyceae and Ulvophyceae. Ulvophyceae was the smallest of the three with just 49 strains and, therefore, random samples of almost the same size (54) from each of the other two classes were used for the statistical analyses. The CVA did not reveal any distinct groups, i.e. the analyzed strains tended to form three groups corresponding to the three green algal classes, but with a considerable overlap among them (Figure 4). However, the three classes were found significantly distinct from each other in both employed significance tests and SIMPER. The latter and correlation analyses allowed to consider 9-octadecanamid (ρ_CV1 _= -0.58/p < 0.001; ρ_CV2 _= -0.22/p < 0.010) and the FA 18:2(9*Z*, 12*Z*) (ρ_CV1 _= -0.44/p < 0.001; ρ_CV2 _= -0.53/p < 0.001) as the only variables to discriminate well Ulvophyceae from Chlorophyceae/Trebouxiophyceae and Trebouxiophyceae from Ulvophyceae/Chlorophyceae, respectively.

##### Generic level analyses

**Figure 5:**
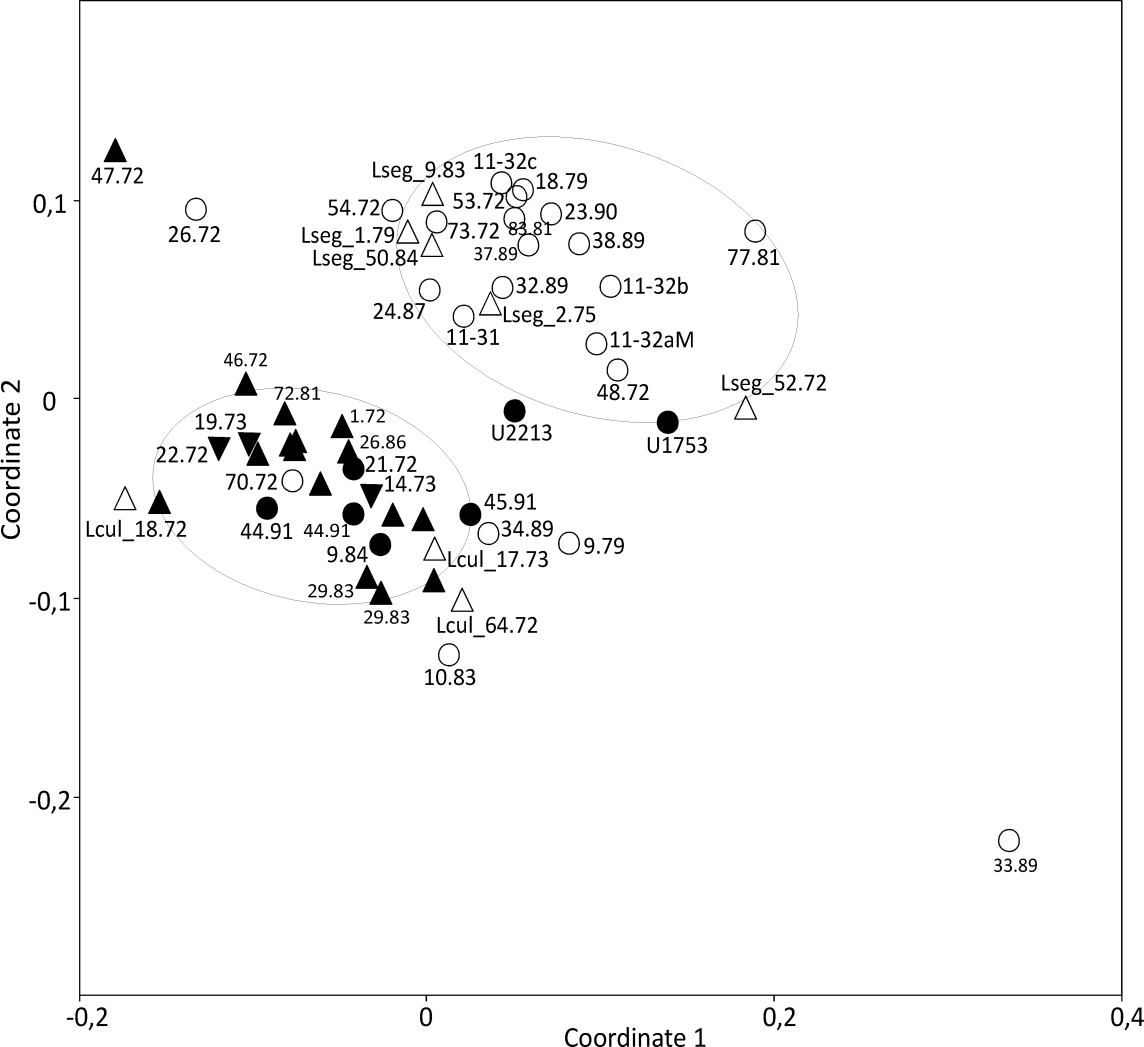
**Distinction of 54 strains previously assigned to *Chlamydomonas s.l*. (Chlorophyceae), into the "*Reinhardtii*" (upper right) and "*Chloromonas*" (lower left) groups as based on fatty acid distribution patterns (non-metric multidimensional scaling, NMDS; Manhattan distance, Kruskal's stress = 0.17)**. Symbols indicate the lineages and genera as resolved in the rDNA analyses of Pröschold et al. (2001); circles, "*Reinhardtii*" clade; empty arrowheads, *Lobochlamys*; filled circles, *Oogamochlamys*; filled arrowheads, *Chloromonas*; filled arrowhead down, "*Moewusii*" clade.

The three previous analyses showed that phylogenetic relationships at the level of phyla and classes among algal groups were reflected in FA distribution patterns using a large sample of strains. Therefore, in a second group of analyses, we tested whether differences in FA distribution patterns may resolve the same distinction of genera as in rRNA gene sequence analyses. To test this, we selected three genera which are widely used in biotechnological applications and well represented by SAG strains, i.e. *Chlorella s.l., Scenedesmus s.l*. and *Chlamydomonas s.l*.. Recent18S rRNA gene sequence analyses revealed each of the three as para- or polyphyletic assemblages encompassing several distinct genera. For *Chlamydomonas *we selected 17 species (53 strains), out of which 9 were represented by multiple strains (e.g., *C. reinhardtii*, 16), which were distributed on five independent lineages/clades (= genera) in the 18S rDNA phylogeny [30]. To better represent the "*Oogamochlamys*" clade also two strains from the UTEX collection (2213, 1753) were included. The NMDS ordination clearly separated the members of the "*Reinhardtii*" clade (upper right in Figure 5), except for three strains, from those of the "*Chloromonas*" clade (lower left in Figure 5). However, the "*Chloromonas*" group as revealed by the FA patterns also included the three investigated strains of the "*Moewusii*" and four of the "*Oogamochlamys*" clades which was in contrast to the 18S rDNA phylogenies of [30]. Also in contrast to the rDNA phylogenies, the FA analyses split the genus *Lobochlamys*, i.e. *L. culleus *was part of the "*Chloromonas*" group while *L. segnis *belonged to the "*Reinhardtii*" group. Strains of *Oogamochlamys *were also separated on both FA groups, in contrast to their species assignments as based on the 18S rDNA analyses.

**Figure 6:**
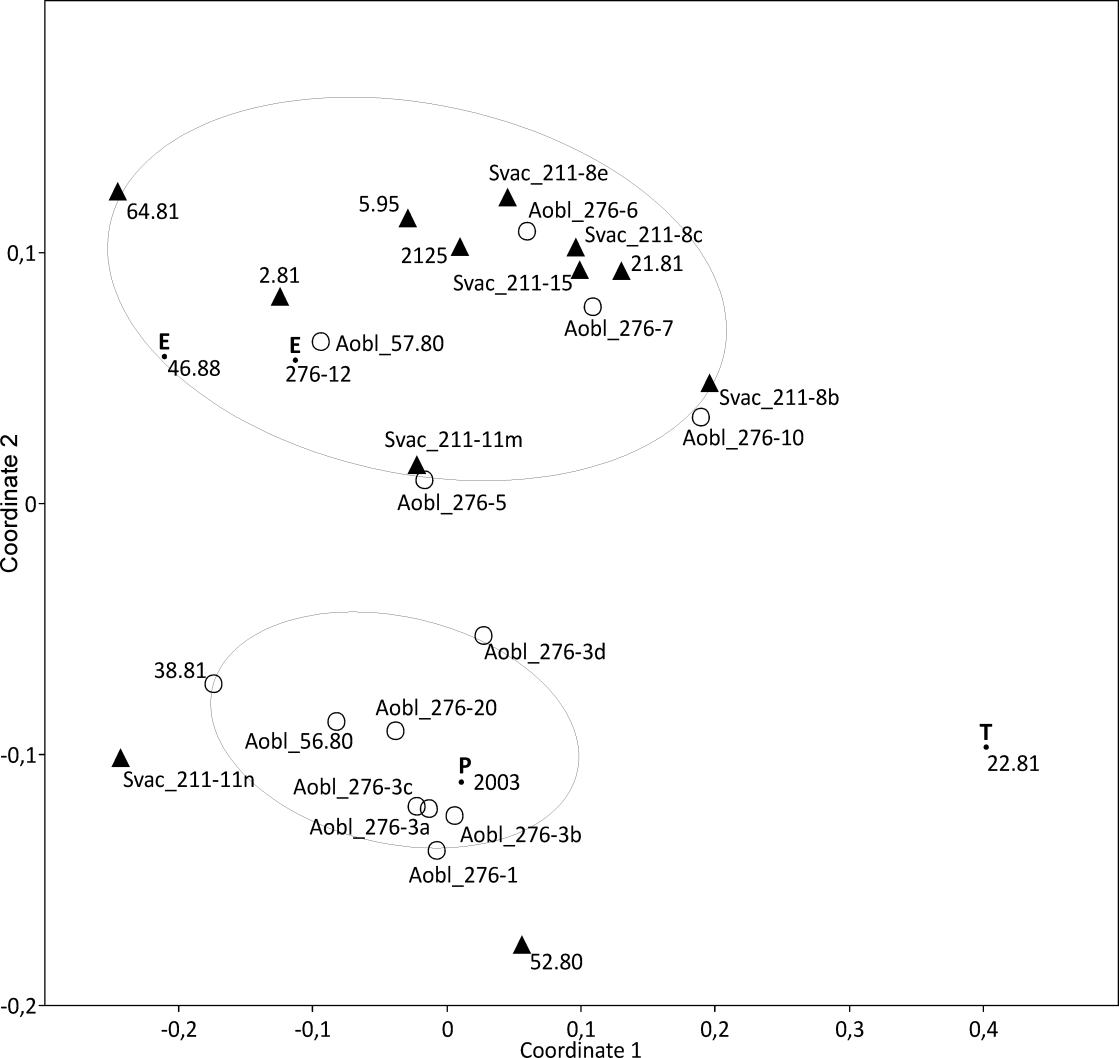
**Separation of *Acutodesmus *(empty circles) from *Scenedesmus s.str*. strains (filled arrowheads) as seen in FA pattern distribution**. Multiple strains of *A. obliquus *are indicated by abbreviation "Aobl", those of *S. vacuolatus *by "Svac". E, P, T, strains of the genera *Enallax, Pectinodesmus *and *Tetradesmus *(Non-metric multidimensional scaling, NMDS; Manhattan distance, Kruskal's stress = 0.16).

Species and strains formerly assigned to a single genus *Scenedesmus *were shown to be actually distributed on several genera by rRNA gene sequence analyses. For example, the genus *Acutodesmus *has been segregated from *Scenedesmus *[31,32]. A NMDS ordination plot of FA distribution patterns revealed a tendency among the studied strains to be distributed on two clusters, i.e. one cluster of 8 strains of *Acutodesmus *(mainly including multiple strains of *A. obliquus*) was clearly separated from another cluster containing mainly strains of *Scenedesmus s.str*. (Figure 6). The multiple strains of *S. vacuolatus *were grouped together with four other strains of the genus, except for SAG 211-11n which was close to the *Acutodesmus *cluster. The multiple strains of *A. obliquus*, however, were distributed on both clusters (Figure 6). Seven strains of *A. obliquus *mainly formed up the *Acutodesmus *cluster, whereas five other *A. obliquus *strains grouped together with strains of *Scenedesmus s.str*. This means that within the same green algal species, *A. obliquus*, two distinct FA patterns exist. AFLP fingerprints already showed extensive genetic variation among the multiple strains of *A. obliquus *while ITS2 rDNA sequence comparisons demonstrated conspecificity of the multiple strains, except for SAG 276-20 (T. Friedl, unpubl. observation). Therefore, the finding of *A. obliquus *strains being separated in two FA pattern groups favours the view that genetic differences resolved by AFLPs may correspond to different phenotypic properties. Consequently, it may be crucial to carefully record which strain has been used in any application [33]. Though strain SAG 276-20 was found not to belong to the same species, *A. obliquus*, its FA pattern suggests that it may still be a member of *Acutodesmus *because it was grouped in the *Acutodesmus *cluster (Figure 6).

**Figure 7:**
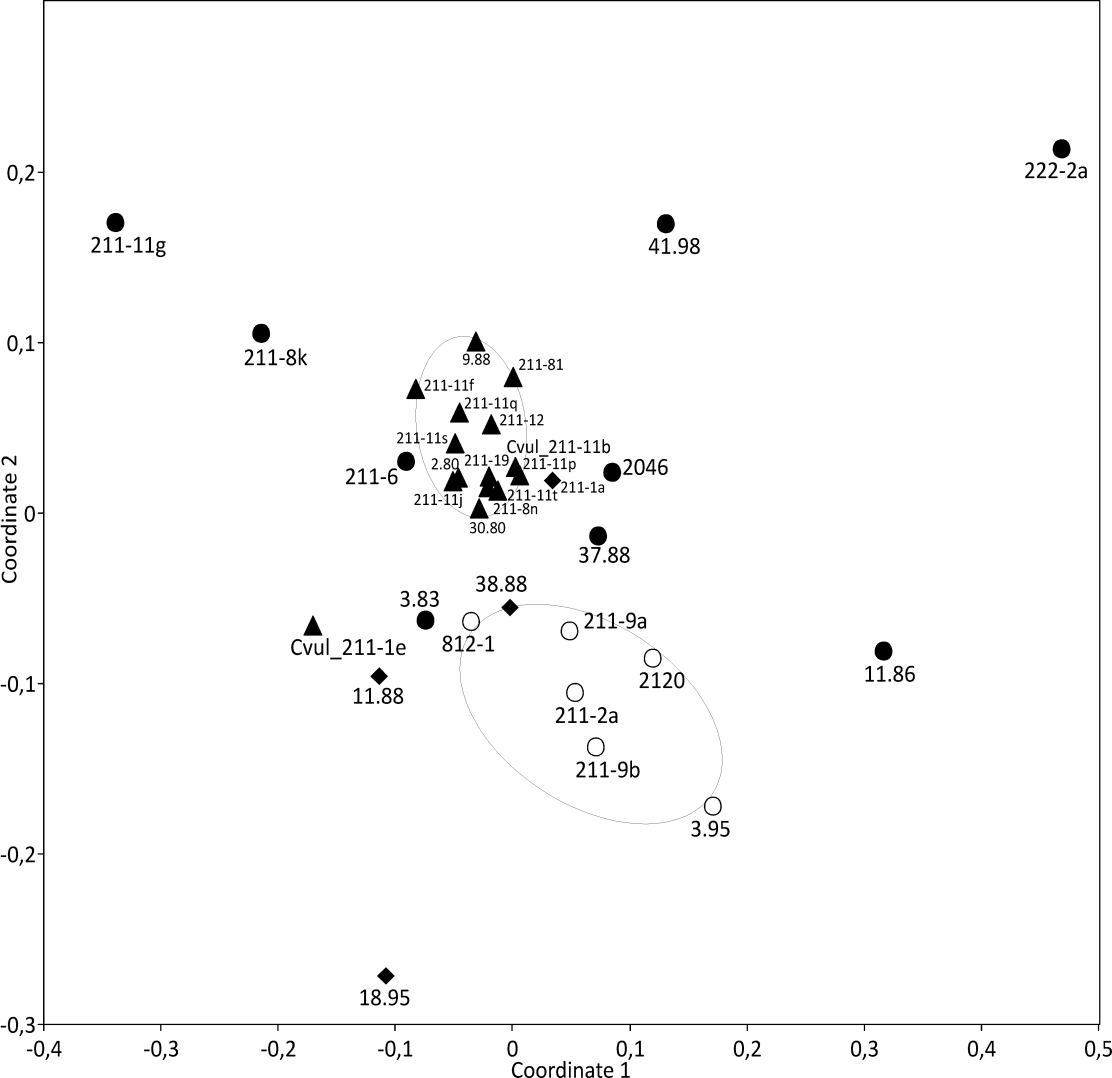
**Comparison of FA patterns of multiple strains of *Chlorella vulgaris *(arrowheads) and their closer relatives (filled circles) with more distantly related *Chlorella-*like green algae of the *Watanabea*- (empty circles) and *Prasiola*-clades (diamonds) *sensu *Darienko et al., 2010 (non-metric multidimensional scaling, NMDS; Manhattan distance, Kruskal's stress = 0.12)**.

*Chlorella vulgaris *forms another example where extensive genetic variation among multiple strains of the same species has been detected by AFLP analyses [33]. The 15 multiple SAG strains of *C. vulgaris *were compared to 19 other *Chlorella *and *Chlorella-*like strains, i.e. their closest relatives as seen in 18S rDNA phylogenies, *C. sorokiniana *and *C. lobophora*, members of the *Parachlorella *clade *sensu *[34] as well as more distantly related strains, i.e. from the *Watanabea *and *Prasiola *clades *sensu *[35]. NMDS ordination based on FA distribution pattern showed almost no variation within the multiple strains of *C. vulgaris *and clustered them together, except for strain SAG 211-1e (Figure 7). Another cluster distant from *C. vulgaris *was formed by members of the *Watanabea*-clade, whereas *Chlorella-*like algae of the *Prasiola*-clade were not clustered together.

## Conclusion

The algae collection at the SAG represents a valuable resource of natural products as shown in the present study for FAs and other hydrophobic metabolites. Several general trends in FA distribution reflect phylogenetic relationships among phyla and classes as seen in genomic and molecular phylogenies and this makes FA distribution patterns an additional feature to define taxa of higher rank in algae. However the FA profile alone may be no useful marker to distinguish among different genera and species. For this, the comparison of further metabolites, like sterols, entire lipids and hydrocarbons should be considered. Thus, PUFA contents in microalgae are rather difficult to predict at the levels of genera and species, making it difficult to select appropriate strains for biotechnological research/applications which aim at yielding high lipid contents. Therefore, each additional or novel isolate will be worth of examination for its PUFA content.

## Methods

### Preparation of microalgal cultures

The microalgal cells were harvested from cultures at the stationary phase and stored at -20°C. Stationary phase was reached after different periods of culturing ranging from three months to about one year, depending on the strain-specific SAG's standard maintenance protocols. Before FA extraction the algal material was lyophilised for two days until the cell pellets were totally dry.

### Alkaline hydrolysis, transesterification and extraction of FA methyl esters (FAMEs)

Prior to FAME extraction the dry weight of lyophilised algal material was determined and then the samples were transferred into a 2 ml tube. The samples were extracted by adding 405 μl of methanol/toluol 2:1 (v/v) followed by homogenisation of the cells with a potter (Heidolph RZR 2020, Schwabach) for 30 s. To avoid autoxidation, the samples were overlaid with argon. As internal standard, 10 μg of tripentadecanoate (diluted in 10 μl toluol) was added. Transesterification of lipid bound FAs to their corresponding FAMEs was accomplished by adding 150 μl sodium methoxide [36]. After 20 min shaking at RT the FAMEs were extracted two times with 500 μl n-hexane and 500 μl 1 M NaCl. The hexane phases were transferred into a 1.5 ml tube and dried under streaming nitrogen. Finally the FAMEs were redissolved in 10 μl acetonitrile and analysed by GC.

### Preparation of 4, 4-dimethyloxaline (DMOX) derivatives

The position of double bonds of unknown FAME isomers was determined by analysing the corresponding DMOX derivatives to allow identification by MS [37]. FAMEs were prepared as described, but the hexane phases were transferred into a 1.5 ml glass tube. Samples were dried under streaming nitrogen and 200 μl 2-alkenyl-4, 4-dimethyloxazoline (Sigma, München) were added. After incubation at 180°C over night in a heating block, the samples were cooled to RT and transferred with 2 ml dichloromethane into a 12 ml glass tube and reextracted with 5 ml hexane and 2 ml water. The hexane phase was dried under streaming nitrogen and redissolved with 50 μl chloroform. The DMOX derivatives were separated on a 20 cm × 20 cm silica gel 60 TLC plate (Merck) with petrol ether/diethyl ether 2:1 (v/v) as a developing solvent. The plate was sprayed with 0.2% 8-anilino-1-naphthalene-sulfonic acid to visualize the DMOX derivatives under UV-light. The blue/yellow band of the DMOX derivatives was scraped out and the derivatives extracted by consecutive addition of 0.4 ml water, 2 ml methanol, 2 ml chloroform and 2 ml saturated NaCl solution. Between each step the sample was vigorously mixed and finally centrifuged for 5 min at 3220 × *g *to separate phases. The lower phase was transferred into a new glass tube and dried under nitrogen stream, redissolved in 10 μl acetonitrile and analysed with GC/MS.

### Identification of FAMEs by GC with flame ionisation detection (FID)

The prepared FAMEs were analysed by GC/FID using a capillary DB-23 column (30 m × 0.25 mm, 0.25 μm coating thickness, J&W, Scientific, Agilent, Waldbronn) according to Hornung et al. (2002). Helium was used as carrier gas with a flow of 0.1 ml/min. The temperature gradient was 150°C for 1 min, 150-200°C at 4 K/min, 200-250°C at 5 K/min and 250°C for 6 min. Tripentadecanoate was added to each sample for quantification and the FAMEs were identified according to the retention time of the corresponding peaks in the standard "F.A.M.E. Mix C4-C24" (Sigma, München), which was injected before every 50^th ^run. The injection volume depended on the concentration of FAMEs within in the sample.

### Identification of FAMEs by GC/MS

FID signals which were not identified by their retention times on GC and either represented FAMEs or other unpolar substances were further analysed by their mass spectra using a 6890 Gas Chromatograph/5973 Mass Selective Detector system (Agilent, Waldbronn). The GC/MS conditions were the same as for GC-analysis. The electron energy was 70 eV, the ion source temperature 230°C, and the temperature for the transfer line added up to 260°C. The identification of unknown substances was done by comparison of the obtained mass spectra with the mass spectra library NIST98 and the "Lipid Library" of the Scottish Crop Science research Institute http://www.lipidlibrary.co.uk/index.html.

### Analysis of FAMEs

All chromatograms of the microalgal samples were analysed by using the ChemStation software version 9.03 (Agilent, Waldbronn). All peaks spanning a peak area of more than 50 units were integrated. The amount of each FAME was calculated using a defined amount (1 μg) of the internal standard tripentadecanoate and the dry weight (DW) of each sample: area of peak × 1 μg/area of tripentadecanoate/mg d.w = μg FAME/mg DW

### Statistical analyses of FA distribution patterns

For each detected fatty acid (FA) its percentage of the total FA content of a strain was used as variable. For the investigation of the general structure of the data sets, common indirect ordination techniques were used, i.e. Principal Components Analysis (PCA), Correspondence and Detrended Correspondence Analysis, and Non-Metric Multidimensional Scaling (NMDS). The significance of the differences among *a priori *predefined algal groups were tested using non-parametric multidimensional significance tests (Non-Parametric Multivariate Analysis of Variance, Analysis of Similarity) and visualised as ordinations from multigroup discriminant analysis (Canonical Variates Analysis). The percentages of dissimilarity between group pairs were investigated conducting SIMPER analysis. To link the significant differences among algal groups with particular variables/fatty acids possibly contributing to the observed difference, correlation analyses were conducted (Spearman's rank correlation coefficient, ρ/rho), permutation significance tests). All statistical analyses and graphical visualisations have been conducted in PAST version 2.07 software package. Final graphical attributes required for publication were adapted in vector graphics editor Inkscape version 4.7 and CorelDraw X3 Graphic suite.

## Authors' contributions

IL carried out the fatty acid analysis of all algal strains and drafted the manuscript. LH performed the statistical analysis. IF and TF conceived of the study, and participated in its design and coordination and helped to draft the manuscript. All authors read and approved the final manuscript.

## List of abbreviations

ALA: α-linolenic acid; ARA: Arachidonic acid; CVA: canonical variance analysis; DHA: docosahexaenoic acid; DMOX: 4, 4-dimethyloxaline; EPA: Eicosapentaenoic acid; FA: fatty acid; FAME: fatty acid methyl ester; GC: gas chromatography; GLA: γ-Linolenic acid; MS: mass spectrometry; NMDS: non-metric multidimensional scaling; PA: palmitic acid; PUFAs: polyunsaturated fatty acids; SAG: culture collection of microalgae in Göttingen; SDA: stearidonic acid.

## Supplementary Material

Additional file 1 **FAME database established of all SAG microalgal strains screened**. The database contains information about clade, phylum, class, genus and species identification (1^st ^to 5^th ^column) as well as SAG strain number (6^th ^column) and the amount of the different substances given as relative proportion (following columns). Click here for file

Additional file 2 **Reduced FAME database for statistical analyses**. The database contains information about clade, phylum, class, genus and species identification (1^st ^to 5^th ^column) as well as SAG strain number (6^th ^column) and the amount of the different substances given as relative proportion (following columns). Click here for file

## References

[B1] Bergé J-P Barnathan G Fatty acids from lipids of marine organisms: Molecular biodiversity, roles as biomarkers, biologically active compounds, and economical aspects Adv Biochem Eng/Biotechnol 2005 96 49 125 10.1007/b135782 16566089

[B2] Dunstan GA Volkman JK Barret SM Leroi J-M Jeffrey SW Essential polyunsaturated fatty acids from 14 species of diatom (*Bacillariophyceae*) Phytochemistry 1994 35 155 161

[B3] Tonon T Harvey D Larson TR Graham IA Long chain polyunsaturated fatty acid production and partitioning to triacylglycerols in four microalgae Phytochemistry 2002 61 15 24 10.1016/S0031-9422(02)00201-7 12165297

[B4] Viso A-C Marty J-C Fatty acids from 28 marine microalgae Phytochemistry 1993 34 1521 1533 10.1016/S0031-9422(00)90839-2

[B5] Harwood JL Guschina IA The versatility of algae and their lipid metabolism Biochimie 2009 91 679 684 10.1016/j.biochi.2008.11.004 19063932

[B6] Watson SB Cyanobacterial and eukaryotic algal odour compounds: signals or by-products? A review of their biological activity Phycologia 2003 42 332 350 10.2216/i0031-8884-42-4-332.1

[B7] Mongrand S Badoc A Patouille B Lacomblez C Chavent M Bessoule J-J Chemotaxonomy of the Rubiaceae family based on leaf fatty acid composition Phytochemistry 2005 66 549 559 10.1016/j.phytochem.2004.12.021 15721947

[B8] Spitzer V Screening analysis of unknown seed oils Fett/Lipid 1999 101 2 19 10.1002/(SICI)1521-4133(19991)101:1<2::AID-LIPI2>3.0.CO;2-H

[B9] Rossi S Sabates A Latasa M Reyes E Lipid biomarkers and trophic linkages between phytoplankton, zooplankton and anchovy (Engraulis encrasicolus) larvae in the NW Mediterranean J Plankton Res 2006 28 551 562 10.1093/plankt/fbi140

[B10] Schweder T Lindequist U Lalk M Screening for new metabolites from marine microorganisms Marine Biotechnology I 2005 96 Springer Berlin/Heidelberg 1 48 *Advances in Biochemical Engineering/Biotechnology* 10.1007/b135781 16566088

[B11] Volkman JK Barrett SM Blackburn SI Mansour MP Sikes EL Gelin F Microalgal biomarkers: A review of recent research developments Org Geochem 1998 29 1163 1179 10.1016/S0146-6380(98)00062-X

[B12] Temina M Rezankova H Rezanka T Dembitsky VM Diversity of the fatty acids of the Nostoc species and their statistical analysis Microbiol Res 2007 162 308 321 10.1016/j.micres.2006.01.010 16563711

[B13] Leblond JD Dahmen JL Seipelt RL Elrod-Erickson MJ Kincaid R Howard JC Evens TJ Chapman PJ Lipid composition of chlorarachniophytes (Chlorarachniophyceae) from the genera *Bigelowiella, Gymnochlora*, and *Lotharella* J Phycol 2005 41 311 321 10.1111/j.1529-8817.2005.04082.x

[B14] Dunstan GA Brown MR Volkman JK Cryptophyceae and Rhodophyceae; chemotaxonomy, phylogeny, and application Phytochemistry 2005 66 2557 2570 10.1016/j.phytochem.2005.08.015 16226285

[B15] Shiran D Khozin I Heimer YM Cohen Z Biosynthesis of eicosapentaenoic acid in the microalga Porphyridium cruentum.1. The use of externally supplied fatty acids Lipids 1996 31 1277 1282 10.1007/BF02587913 8972461

[B16] Cohen Z The production potential of eicosapentaenoic and arachidonic acids by the red alga *Porphyridium cruentum* J Am Oil Chem Soc 1990 67 916 920 10.1007/BF02541847

[B17] Zook D Schenk HEA Lipids in cyanophora paradoxa. III. Lipids in cell compartments Endocyt C Res 1986 3 203 211

[B18] Cavalier-Smith T The origin of eukaryotic and archaebacterial cells Ann N Y Acad Sci 1987 503 17 54 10.1111/j.1749-6632.1987.tb40596.x 3113314

[B19] Neustupa J Elias M Skaloud P Nemcova Y Sejnohova L *Xylochloris irregularis *gen. et sp. nov. (Trebouxiophyceae, Chlorophyta), a novel subaerial coccoid green alga Phycologia 2011 50 57 66 10.2216/08-64.1

[B20] Bigogno C Khozin-Goldberg I Boussiba S Vonshak A Cohen Z Lipid and fatty acid composition of the green oleaginous alga Parietochloris incisa, the richest plant source of arachidonic acid Phytochemistry 2002 60 497 503 10.1016/S0031-9422(02)00100-0 12052516

[B21] Caudales R Wells JM Differentiation of free-living *Anabaena *and *Nostoc *cyanobacteria on the basis of fatty-acid composition Int J Syst Bacteriol 1992 42 246 251 10.1099/00207713-42-2-246 1581185

[B22] Krüger G De Wet H Kock J Pieterse A Fatty acid composition as a taxonomic characteristic for *Microcystis *and other coccoid cyanobacteria (blue-green alga) isolates Hydrobiologia 1995 308 145 151 10.1007/BF00007400

[B23] Dembitsky VM Srebnik M Variability of hydrocarbon and fatty acid components in cultures of the filamentous cyanobacterium Scytonema sp. isolated from microbial community "black cover" of limestone walls in Jerusalem Biochemistry (Mosc) 2002 67 1276 1282 10.1023/A:1021309623541 12495426

[B24] Kenyon CN Fatty acid composition of unicellular strains of blue-green algae J Bacteriol 1972 109 827 834 4621688 10.1128/jb.109.2.827-834.1972 PMC285212

[B25] Kenyon CN Stanier RY Possible evolutionary significance of polyunsaturated fatty acids in blue-green algae Nature 1970 227 1164 1166 10.1038/2271164a0 4988959

[B26] Keeling PJ Diversity and evolutionary history of plastids and their hosts Am J Bot 2004 91 1481 1493 10.3732/ajb.91.10.1481 21652304

[B27] Gould SB Waller RF McFadden GI Plastid evolution Annu Rev Plant Biol 2008 59 491 517 10.1146/annurev.arplant.59.032607.092915 18315522

[B28] Friedl T The evolution of the green algae Plant Syst Evol 1997 11 87 101

[B29] Lewis LA McCourt RM Green algae and the origin of land plants Am J Bot 2004 91 1535 1556 10.3732/ajb.91.10.1535 21652308

[B30] Pröschold T Marin B Schlosser UG Melkonian M Molecular phylogeny and taxonomic revision of Chlamydomonas (*Chlorophyta*). I. Emendation of Chlamydomonas Ehrenberg and Chloromonas Gobi, and description of Oogamochlamys gen. nov. and Lobochlamys gen. nov Protist 2001 152 265 300 10.1078/1434-4610-00068 11822658

[B31] Elias M Nemcova Y Skaloud P Neustupa J Kaufnerova V Sejnohova L *Hylodesmus singaporensis *gen. et sp. nov., a new autosporic subaerial green alga (Scenedesmaceae, Chlorophyta) from Singapore Int J Syst Evol Microbiol 2010 60 1224 1235 10.1099/ijs.0.012963-0 19666799

[B32] Hegewald E Wolf M Phylogenetic relationships of Scenedesmus and Acutodesmus (Chlorophyta, Chlorophyceae) as inferred from 18S rDNA and ITS-2 sequence comparisons Plant Syst Evol 2003 241 185 191 10.1007/s00606-003-0061-7

[B33] Müller J Friedl T Hepperle D Lorenz M Day JG Distinction between multiple isolates of *Chlorella vulgaris *(Chlorophyta, Trebouxiophyceae) and testing for conspecificity using Amplified Fragment Length Polymorphism and its rDNA sequences J Phycol 2005 41 1236 1247 10.1111/j.1529-8817.2005.00134.x

[B34] Krienitz L Hegewald EH Hepperle D Huss VAR Rohrs T Wolf M Phylogenetic relationship of *Chlorella *and *Parachlorella *gen. nov (Chlorophyta, Trebouxiophyceae) Phycologia 2004 43 529 542 10.2216/i0031-8884-43-5-529.1

[B35] Pröschold T Darienko T Gustavs L Mudimu O Menendez CR Schumann R Karsten U Friedl T Chloroidium, a common terrestrial coccoid green alga previously assigned to Chlorella (Trebouxiophyceae, Chlorophyta) Eur J Phycol 2010 45 79 95 10.1080/09670260903362820

[B36] Hornung E Pernstich C Feussner I Formation of conjugated Δ^11 ^Δ^13^-double bonds by Δ^2^-linoleic acid (1, 4)-acyl-lipid-desaturase in pomegranate seeds Eur J Biochem 2002 269 4852 4859 10.1046/j.1432-1033.2002.03184.x 12354116

[B37] Fay L Richli U Location of double bonds in polyunsaturated fatty acids by gas chromatography-mass spectrometry after 4, 4-dimethyloxazoline derivatization J Chromatogr A 1991 541 89 98

